# The Keystone commensal bacterium *Christensenella minuta* DSM 22607 displays anti-inflammatory properties both in vitro and in vivo

**DOI:** 10.1038/s41598-021-90885-1

**Published:** 2021-06-01

**Authors:** Camille Kropp, Katy Le Corf, Karima Relizani, Kevin Tambosco, Ccori Martinez, Florian Chain, Georges Rawadi, Philippe Langella, Sandrine P. Claus, Rebeca Martin

**Affiliations:** 1 Micalis Institute, AgroParisTech, INRAE, Université Paris-Saclay, 78350 Jouy-en-Josas, France; 2 Ysopia Bioscience, 33000 Bordeaux, France

**Keywords:** Bacteriology, Bacterial host response

## Abstract

*Christensenellaceae* is a family of subdominant commensal bacteria found in humans. It is thought to play an important role in gut health by maintaining microbial symbiosis. Indeed, these bacteria occur at significantly lower levels or are absent in individuals suffering from inflammatory bowel diseases (IBDs). Here, we explored if type species *Christensenella minuta* (strain: DSM 22607) could have the potential to help treat IBDs. We assessed key properties displayed by the bacterium using a combination of in vitro and in vivo assays. We found that while *C. minuta* is a strict anaerobe, it is also oxygen tolerant. Additionally, we observed that the species produces high levels of acetate and moderate levels of butyrate. We performed deep phenotyping using Biolog microarrays. Using human intestinal cell lines, we discovered that *C. minuta* demonstrated strong anti-inflammatory activity, resulting in reduced levels of proinflammatory IL-8 cytokines via the inhibition of the NF-*κ*B signaling pathway. Furthermore, *C. minuta* protected intestinal epithelial integrity in vitro. Finally, in two distinct animal models of acute colitis, *C. minuta* prevented intestinal damage, reduced colonic inflammation, and promoted mucosal healing. Together, these results indicate that *C. minuta* has potent immunomodulatory properties, underscoring its potential use in innovative microbiome-based IBD biotherapies.

## Introduction

Inflammatory bowel diseases (IBDs) are disorders characterized by the chronic abnormal inflammation of the gastrointestinal tract, which triggers an uncontrolled and deleterious inflammatory response^1,2^ during which levels of interleukine-8 (IL-8) cytokines increase^3,4^ and reactive oxygen species (ROS) are overproduced^5^. The two main types of IBDs are Crohn's disease (CD) and ulcerative colitis (UC)^6^, which each display distinct physiological symptoms. Although the aetiology of IBDs remains poorly understood, we do know that a complex set of interconnected environmental^7^, genetic^8^, and immune^9^ factors are involved. IBD triggers are also little characterized, but the best-supported hypothesis is that immune system disruptions provoke imbalances in crosstalk between gut commensal bacteria and human hosts^2^. Indeed, in multiple studies, individuals suffering from CD or UC have been found to display microbial dysbiosis, characterized by a decrease in commensal bacteria in the phyla *Firmicutes* and *Bacteroidetes,* allowing an increase in bacteria in the class *Gammaproteobacteria*^10–13^. This dysbiosis is accompanied by changes in short-chain fatty acid (SCFA) production^14,15^, which can affect inflammation pathways and immune system modulation^14^.

Because proper balance within the intestinal microbiota helps ensure health, strategies have been developed to address IBD-related dysbiosis. To date, fecal microbiota transplantation (FMT)^16^ and treatments using isolated bacteria^17^ have yielded promising results. FMT has been shown to be efficient in treating *Clostridium difficile* infections^18^; it may also help restore the microbiota of those suffering from IBDs, but more research is needed to support its routine use in IBD cases^19^. Indeed, this procedure can be difficult to set up and control. Donor choice is an especially important consideration^20^ because donor incompatibility can lead to alterations in nutrient absorption, promote the onset of chronic disease, or transfer undesired microorganisms^21^. Recently, in murine models, treatments employing single bacterial strains have been successfully used to modulate gut microbial populations^22^ and restore gastrointestinal health by reducing tissue damage and levels of pro-inflammatory cytokines^12,23–26^. Moreover, in humans, these approaches can correct dysbiosis and decrease quantities of inflammatory mediators, leading to remission^27^.

In 2017, research showed that the recently described family *Christensenellaceae* plays a major role in gut health^28^: these bacteria serve as keystone species in the development of the symbiotic gut microbiome. *Christensenella* is part of the phylum Firmicutes and the order Clostridiales, and its members are strict anaerobic, Gram-negative, non-sporulating, and non-motile bacteria^29^. This family has been described as a highly heritable taxon and serves as a hub in a co-occurrence network that includes other heritable taxa^30^. Studies have found that individuals suffering from CD or UC have significantly lower levels of or completely lack *Christensenellaceae* in their intestinal microbiota^31,32^. Furthermore, in individuals with CD, such decreases in abundance are highly predictive of flare-ups^33^. Recently, it was discovered that IBD-related alterations in gut microbiota contribute to inflammation dynamics as well as the loss of commensal bacteria that are key to restoring balance and general homeostasis^34^. Taken together, these findings suggest a strong link exists between the abundance of *Christensenellaceae* and the occurrence of IBDs, indicating that these commensal bacteria could play a central role in gut physiology.

Here, we hypothesized that *Christensenella* species have anti-inflammatory properties that protect the intestinal mucosa. To test this hypothesis, we first characterized various properties displayed by the type species *Christensenella minuta* DSM 22607, namely its oxygen sensitivity, metabolic profile, and ability to produce SCFAs. Then, in vitro, we assessed how well the bacterium modulated inflammation in human colonic cells and protected intestinal barrier integrity during inflammation. Finally, we explored whether *C. minuta* DSM 22607 could reduce inflammation in vivo in two distinct preclinical colitis models in rodents. We found support for our hypothesis—this bacterial species may hold promise for microbiome-based IBD biotherapies.

## Results

### An oxygen tolerant anaerobe that produces short-chain fatty acids

**Figure 1:**
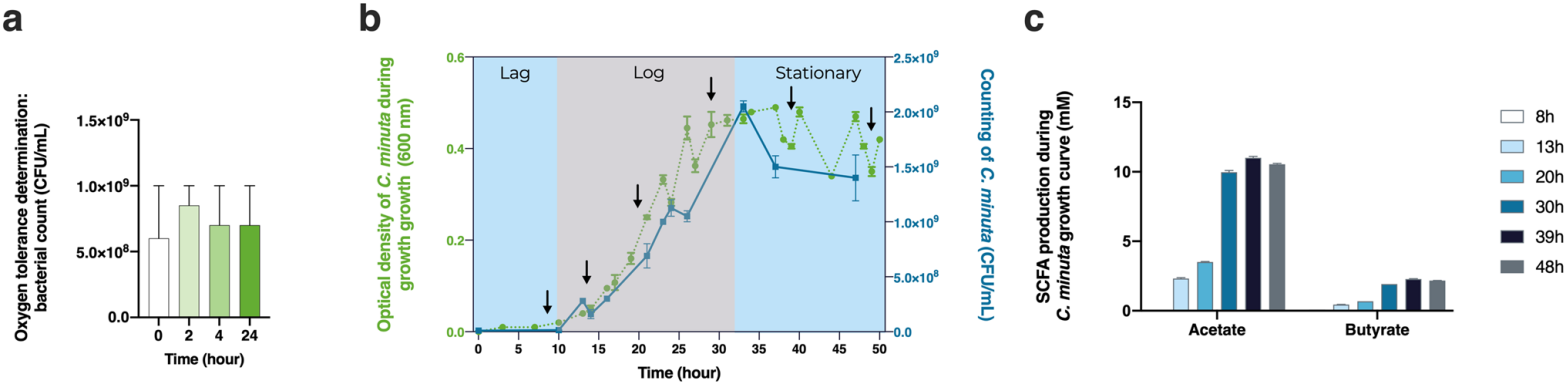
Oxygen sensitivity and metabolite production in *Christensenella minuta*. (**a**) Bacterial counts (CFUs/mL) after 0, 2, 4, and 24 h of oxygen exposure. (**b**) Growth over time expressed in terms of optical density and colony forming units (CFUs). The arrows show the different sampling points. (**c**) Butyrate and acetate production at different sampling points.

To better characterize *C. minuta* DSM 22607, we first assessed the strain’s oxygen sensitivity. We found that the bacterium was not extremely oxygen sensitive (EOS) because it could tolerate the presence of oxygen for at least 24 h (Fig. 1a). We then quantified its production of SCFAs during different growth phases (latent, exponential, and stationary) (Fig. 1b,c). We observed that *C. minuta* was able to produce high levels of acetate and moderate levels of butyrate, as also seen elsewhere^29^; in contrast, no propionate was generated. Our results show that, during all three growth phases, SCFA production ratios remained the same: approximately 1 mol of butyrate for each 5 mol of acetate.

#### The bacterium’s detailed metabolic phenotype

We obtained an extensive phenotypic profile for *C. minuta* using Biolog microassays. We performed three independent replicates. We found that *C.* minuta can metabolize *N*-acetyl-d-glucosamine, d-arabitol, arbutin, d-cellobiose, dextrin, d-fructose, l-fucose, d-galactose, α-d-glucose, maltotriose, d-mannitol, d-mannose, 3-methyl-d-glucose, palatinose, salicin, turanose, fumaric acid, pyruvic acid, l-phenylalanine, 2′-deoxyadenosine, inosine, and uridine (Table 1).

**Table 1:** Metabolic profile of *Christensenella minuta strain DSM 22607.*

Target compound	*C. minuta*	Target compound	*C. minuta*	Target compound	*C. minuta*
Water	−	Turanose	+	3-Methyl-d-glucose	+
*N*-Acetyl-d-galactosamine	−	Acetic acid	−	α-Methyl-d-galactoside	−
*N*-Acetyl-d-glucosamine	+	Formic acid	−	β-Methyl-d-galactoside	−
*N*-Acetyl-β-d-mannosamine	−	Fumaric acid	+	α-Methyl-d-glucoside	−
Adonitol	−	Glyoxylic acid	−	β-Methyl-d-glucoside	–
Amygdalin	−	α-Hydroxybutyric acid	−	Palatinose	+
d-Arabitol	+	β-Hydroxybutyric acid	−	d-Raffinose	−
Arbutin	+	Itaconic acid	−	l-Rhamnose	−
d-Cellobiose	+	α-Ketobutyric acid	–	Salicin	+
α-Cyclodextrin	−	α-Ketovaleric acid	−	d-Sorbitol	−
β-Cyclodextrin	−	D,l-Lactic Acid	−	Stachyose	−
Dextrin	+	l-Lactic acid	−	Sucrose	−
Dulcitol	−	d-Lactic acid methyl ester	−	d-Trehalose	−
i-Erythritol	−	d-Malic acid	−	Glycyl-l-aspartic acid	−
d-Fructose	+ +	l-Malic acid	−	Glycyl-l-glutamine	−
l-Fucose	+ +	Propionic acid	−	Glycyl-l-methionine	−
d-Galactose	+	Pyruvic acid	+	Glycyl-l-proline	−
d-Galacturonic acid	–	Pyruvic acid methyl ester	−	l-Methionine	−
Gentiobiose	−	d-Saccharic acid	−	l-Phenylalanine	+
d-Gluconic acid	−	Succinamic acid	−	l-Serine	−
d-Glucosaminic acid	–	Succinic acid	−	l-Threonine	−
α-d-Glucose	+ +	Succinic acid monomethyl ester	−	l-Valine	–
Glucose-1-phosphate	−	m-Tartaric acid	−	l-Valine plus l-aspartic acid	–
Glucose-6-phosphate	−	Urocanic acid	−	2′-Deoxyadenosine	+
Glycerol	−	l-Alaninamide	−	Inosine	+
D, l-α-Glycerol phosphate	−	l-Alanine	−	Thymidine	−
m-Inositol	−	l-Alanyl-l-glutamine	−	Uridine	+
α-d-Lactose	−	l-Alanyl-l-histidine	−	Thymidine-5′-monophosphate	−
Lactulose	–	l-Alanyl-l-threonine	−	Uridine-5′-monophosphate	−
Maltose	−	l-Asparagine	−	d-Mannose	+ +
Maltotriose	+	l-Glutamic acid	−	d-Melezitose	−
d-Mannitol	+ +	l-Glutamine	−	d-Melibiose	−

### An ability to limit IL-8 production and inhibit NF-κB activation in HT-29 cells

**Figure 2:**
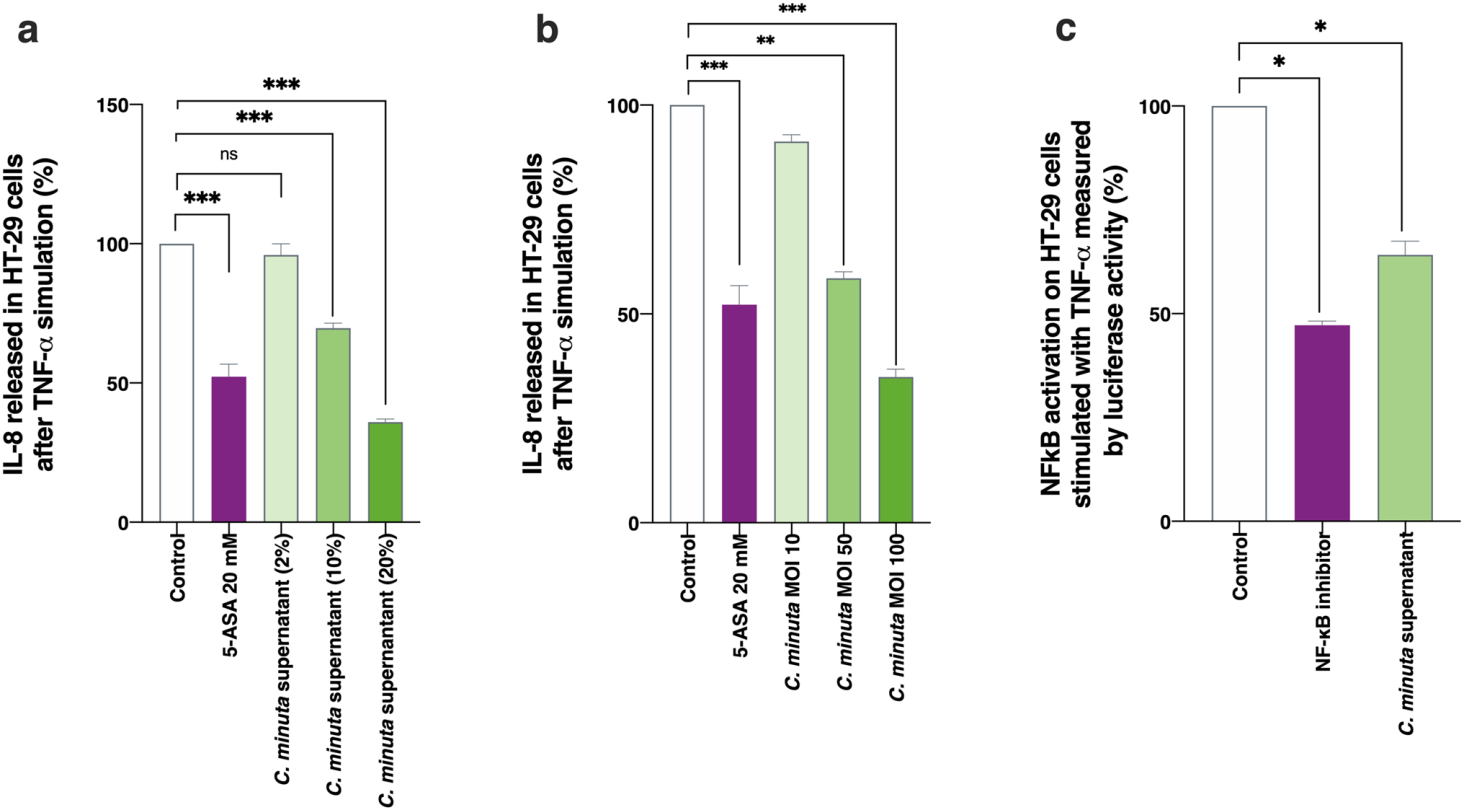
Anti-inflammatory properties of *Christensenella minuta *in vitro. IL-8 production by HT-29 cells exposed to TNF-α in presence of (**a**) *C. minuta* supernatant or (**b**) *C. minuta* bacteria. (**c**) Levels of NF-κB activation in HT-29 cells transfected with a reporter system and exposed to TNF-α. Results of Mann Whitney U tests comparing the control groups to the other groups: *p < 0.05, **p < 0.01, and ***p < 0.001.

Using human HT-29 cells, we analyzed how well *C. minuta* and its supernatant could modulate TNF-α induced secretion of IL-8 and thus limit inflammation. IL-8 is a major proinflammatory cytokine, and, therefore, bacteria capable of inhibiting its secretion have anti-inflammatory effects^35^. Both the supernatant (Fig. 2a) and the bacterium itself (Fig. 2b) demonstrated anti-inflammatory properties: IL-8 production decreased by around 50% in both cases. This level of inhibition resembles that associated with 5-ASA, a compound commonly used to treat IBDs. When co-incubation occurred at different multiplicities of infection (MOIs), we observed a dose–response effect, where percent inhibition ranged from 0% (MOI 10) to 50% (MOI 50) (Fig. 2b). Similar results were obtained with 2% (*v/v*), 10% (*v/v*), and 20% (*v/v*) of the supernatant. We also explored *C. minuta*’s effects on the NF-κB pathway, which plays a key role in inflammation by regulating immune responses^36^, including IL-8 production^37^. The bacterial supernatant decreased NF-κB activation by 40%, an effect similar to that of the control NF-κB inhibitor BAY 11-7082 (10 µm) (Fig. 2c). In contrast, no effects were observed when the bacterium alone was used (data not shown). We thus concluded that *C. minuta* is likely secreting a potent anti-inflammatory effector into its culture medium.

### An ability to maintain barrier integrity in Caco-2 cells

**Figure 3:**
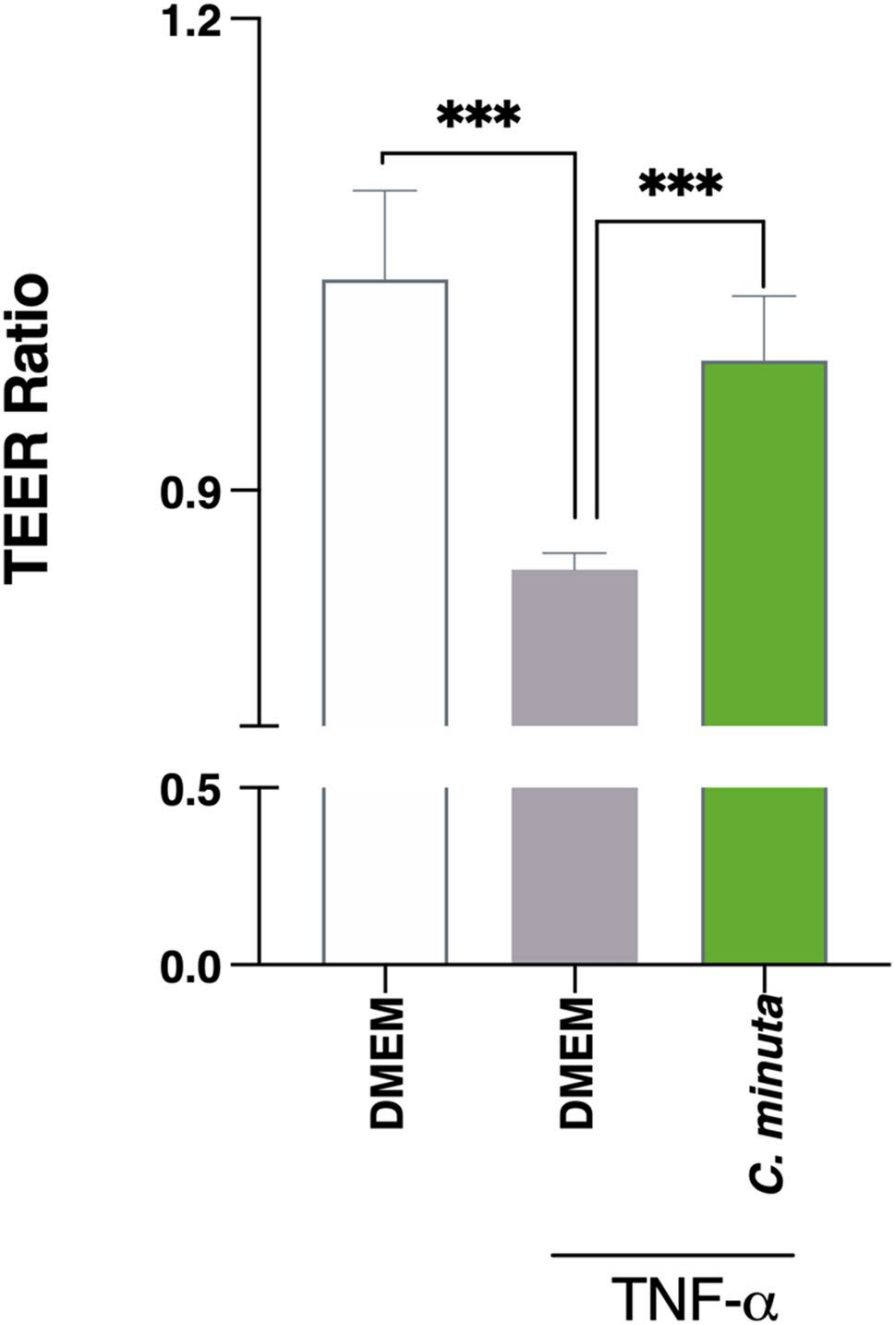
Effects of *Christensenella minuta* on intestinal barrier permeability. Polarized monolayers of Caco-2 cells were exposed to TNF-α to disrupt the intestinal barrier. TEER was measured immediately prior to and 24 h after TNF-α exposure. Results of Mann Whitney U tests comparing the DMEM + TNF-α group to the other groups: *p < 0.05, **p < 0.01, and ***p < 0.001.

We assessed whether *C. minuta* could maintain intestinal barrier integrity in an in vitro cell model by measuring transepithelial electrical resistance (TEER) in Caco-2 cells exposed to TNF-α, which disrupts tight junctions, increases epithelial barrier permeability, and causes inflammation. Measurements were made immediately prior to and 24 h after TNF-α exposure. We observed that the TEER ratio remained stable when Caco-2 cells were treated with *C. minuta* in Dulbecco's modified Eagle’s medium (DMEM) for 3 h beforehand (Fig. 3). This result indicates that barrier integrity had been maintained, seemingly via the anti-inflammatory action of different effectors that protected the intestinal barrier.

### A faculty to prevent and protect against DNBS-induced colitis

**Figure 4:**
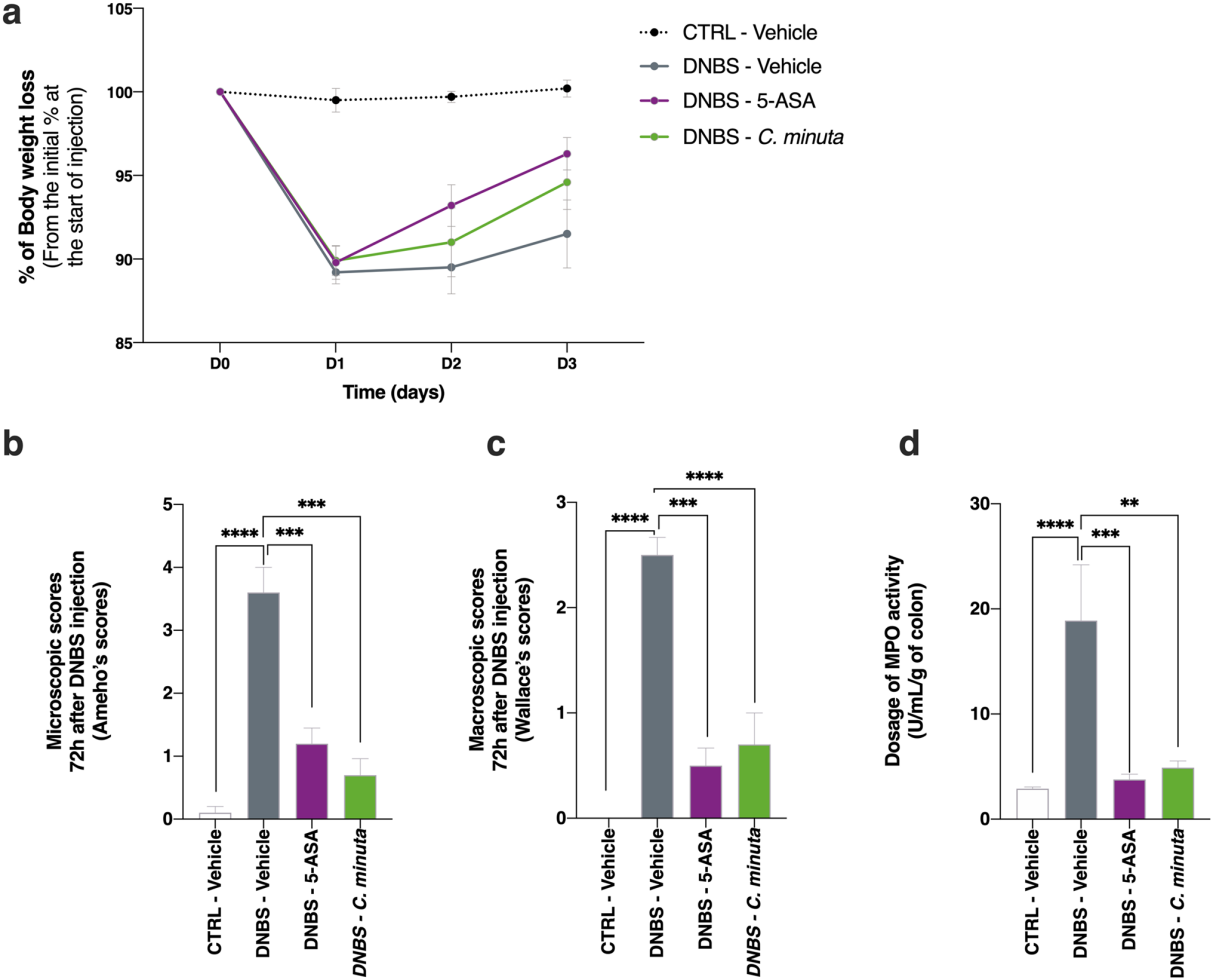
Effects of *Christensenella minuta* on DNBS-induced inflammation in mice. (**a**) Change in body mass after colitis was induced; (**b**) colon microscopic scores; (**c**) colon macroscopic scores; and (**d**) levels of MPO activity. Results of Mann Whitney U tests comparing the DNBS-Vehicle group to the three other groups: *p < 0.05, **p < 0.01, ***p < 0.001, and ****p < 0.0001.

We performed an experiment to determine whether the anti-inflammatory properties of *C. minuta* seen in vitro were also observed in vivo. Treatment mice were given daily doses of *C. minuta* for 14 days. Colitis was then induced by an intrarectal injection of DNBS, and the mice were euthanized 3 days later. We found that the treatment group tended to gain body mass faster than the DNBS-vehicle group (Fig. 4a), although this difference was not significant. We also observed a decrease in the microscopic scores in the treatment group, reflecting restored colonic epithelial structure and reduced immune cell infiltration (Fig. 4b). A similar pattern was seen in the macroscopic scores (Fig. 4d). To evaluate the bacterium’s anti-inflammatory effects on colonic tissue, we characterized the activity of myeloperoxidase (MPO), an enzyme found in the intracellular granules of neutrophils^38^. We observed that DNBS-induced inflammation resulted in increased neutrophil infiltration and MPO activity; these effects were significantly less pronounced in the treatment group gavaged with *C. minuta* (Fig. 4e).

### An ability to prevent and protect against TNBS-induced colitis

**Figure 5:**
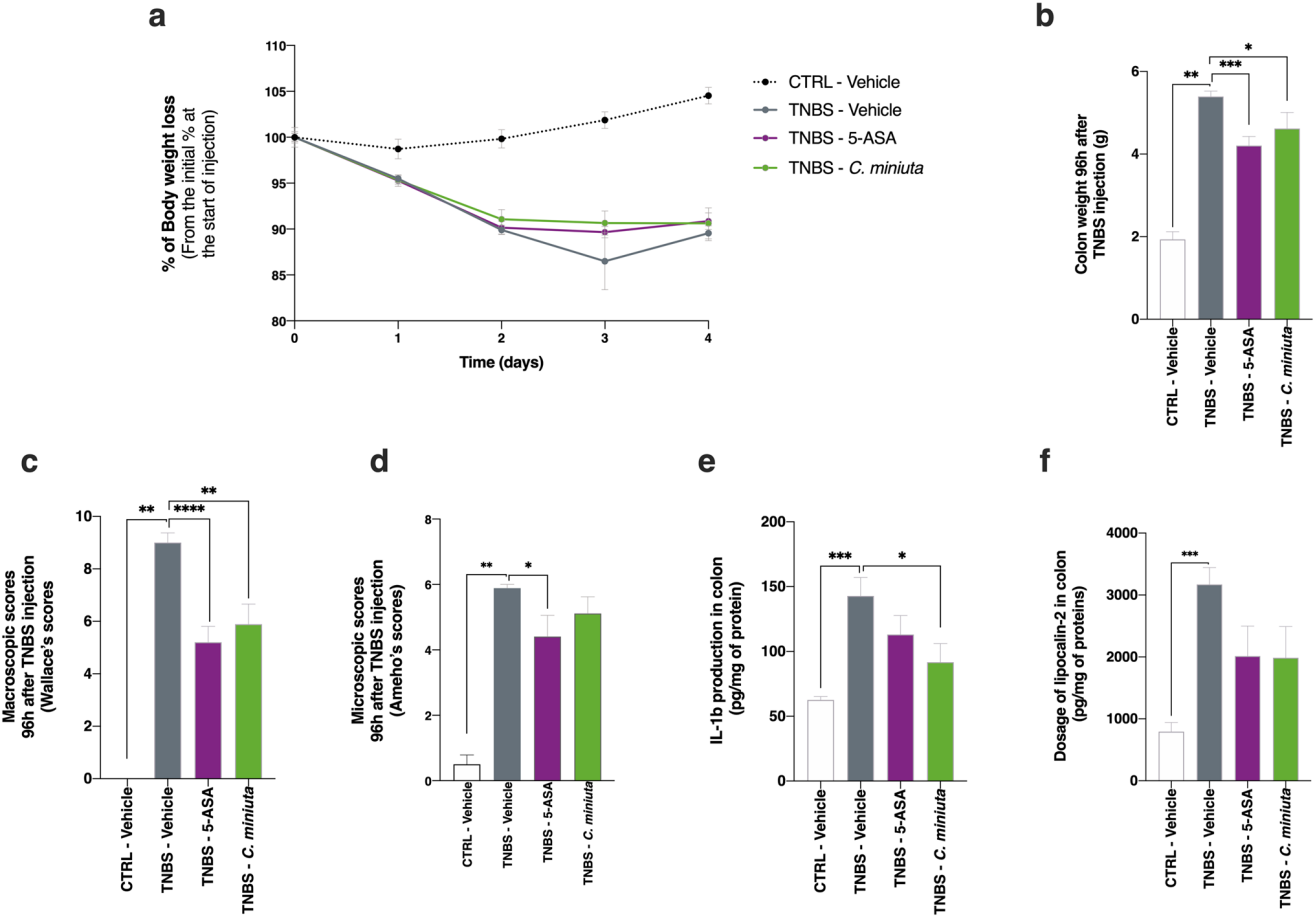
Effects of *Christensenella minuta* on TNBS-induced inflammation in rats. (**a**) Body mass over the course of the experiment; (**b**) colon mass at the end of the experiment; (**c**) colon microscopic scores; (**d**) colon macroscopic scores; colon IL-1β levels (**e**); and (**f**) levels of LCN-2, a proxy for colon neutrophil infiltration. Results of Mann Whitney U tests comparing the TNBS-Vehicle group to other three groups: *p < 0.05, **p < 0.01, ***p < 0.001, and ****p < 0.0001.

To obtain additional confirmation of the bacterium’s anti-inflammatory effects in vivo, we repeated the experiment in a second model—TNBS-induced colitis in rats, known to be more susceptible to inflammation^39^. Treatment rats were given daily doses of *C. minuta* for 14 days. Colitis was then induced by an intrarectal injection of TNBS, and the rats were euthanized 4 days later. Unlike the the mouse model, the treatment group had no effect on body mass gain, compared to TNBS-vehicle group at the end of the experiment (Fig. 5a). However, colon mass was lower in the treatment group (Fig. 5b), which indicates that intestinal transit was improved by *C. minuta*. Remarkably, the macroscopic scores (i.e., Wallace scores^40^; Fig. 5c) provided support for the idea that *C. minuta* could be as efficient as 5-ASA, a compound used to treat UC^41^, in protecting colonic tissue. Moreover, the microscopic scores for the treatment group showed that inflammatory profiles seemed to be improved at the histological level (Fig. 5d), compared to what was seen in the TNBS-vehicle group. Furthermore, the *C. minuta* treatment appeared to induce an immunomodulatory response by decreasing IL-1β secretion (Fig. 5e). This result specifically indicates that the TNBS-induced Th1 response was dampened. IL-6 and IL-10 production (of Th2 and Th1 cytokines, respectively) was not affected by the TNBS injection (data not shown). Finally, we used lipocalin-2 (LCN-2) as a non-invasive biological marker of intestinal inflammation. The *C. minuta* treatment tended to decrease the concentration of LCN-2 in the colon (Fig. 5f). These results validated our in vitro findings, demonstrating the bacterium’s anti-inflammatory properties in two in vivo colitis models.

## Discussion

IBDs are debilitating chronic diseases for which no curative treatments are currently available. Our research is grounded in the idea that microbiome-based therapies offer an innovative approach to healing intestinal mucosa. Indeed, there is increasing evidence that some commensal bacteria possess anti-inflammatory properties that can improve IBD symptoms^10–12^.

In 2012, Morotomi et al.^42^ described a new family of bacteria, the *Christensenellaceae*, whose presence was later found to be correlated with gut microbiota health^28^. Although this taxon is a subdominant member of the microbiome, co-occurrence analyses have revealed that it plays a central role within a broader network of heritable bacteria in the gut ecosystem^30,43^. Indeed, individuals with IBDs display drastically lower levels of these bacteria in their intestines, suggesting that intestinal inflammation is greater when the abundance of *Christensenella* species is lower^31,44,45^. Consequently, we explored the anti-inflammatory properties of *Christensenella minuta* (strain DSM 22607) to determine whether it could be used in IBD treatments.

First, we characterized the bacterium’s metabolic phenotype to gain insight into its overall metabolic capacities. We found that, while *C. minuta* is an anaerobe, it was highly tolerant of oxygen, unlike other anaerobic commensal bacteria, such as *Faecalibactium prausnitzii,* which are EOS. Indeed, recent research has discovered that *Christensenella* occurs in different parts of the human intestine that vary in oxygen concentrations (i.e., the terminal ileum^13,44^, cecum^46^, and distal colon^47^). These findings support the idea that *C. minuta* could have beneficial effects within the upper gastrointestinal tract and, particularly, in the distal ileum, a major site of inflammation in Crohn’s disease; in contrast, other candidate EOS bacteria only occur in the colon. Since IBDs are associated with oxidative stress and high levels of ROS^5^, the ability of *C. minuta* to tolerate oxygen might confer resistance to inflammation-induced oxidative stress in the gut. The bacterium might thus be well suited to creating environmental conditions that allow the establishment of more sensitive anaerobes species. Indeed, the increasing presence of facultative anaerobes observed in the colon during IBD^48^ could give a major advantage to *C. minuta* as a biotherapy against EOS candidates. The bacterium’s oxygen tolerance could also facilitate its use in industrial manufacturing processes, a practical consideration if *C. minuta*’s benefits are to be translated into microbiome-based clinical treatments.

We also confirmed that *C. minuta* produces high levels of acetate and moderate levels of butyrate^29,49^ and demonstrated that the acetate:butyrate production ratio was 5:1 over all three growth phases. Widely produced by gut bacteria, SCFAs result from carbohydrate fermentation and, to a lesser extent, from protein fermentation^50^. Interestingly, a number of bacteria have been identified as either acetate or butyrate producers but rarely both^51^. SCFAs are crucial compounds since they modulate host pathways through interactions with G-protein-coupled receptors (GPRs), which are found in colonic, hepatic, muscular, and adipose tissues^52^. These interactions influence multiple important functions related to cell differentiation and energy metabolism. The butyrate receptor GPR109a also occurs in intestinal epithelial cells, adipocytes, and immune cells, where it helps control inflammation and cell proliferation^53^. Decreases in SCFA production could have deleterious effects, mainly by influencing host-microbe interactions^34^. Consequently, balanced SCFA production is essential to gut homeostasis.

Because SCFAs can affect microbiota-host crosstalk via their immunomodulatory properties^54^, we ascertained whether *C. minuta* had an influence on inflammation. We discovered that both *C. minuta* bacteria and their its supernatant displayed potent anti-inflammatory properties, decreasing the secretion of IL-8 cytokines by HT-29 cells following TNF-α induced inflammation. Such anti-inflammatory properties have also been seen in other bacteria, including *Faecalibacterium prausnitzii*^55^, several strains of *Lactobacillus*^56^, and *Akkermensia muciniphila*^24^*.*

To further explore the bacterium’s anti-inflammatory effects, we tested the impact of both the bacteria and its supernatant against HT-29 cells transfected with a NF-κB reporter system known to regulate IL-8 production^37^. Only the supernatant decreased NF-κB activation. This result, combined with the findings of the previous experiment, suggest that at least two different bacterial effectors were responsible for the effects observed. Past work using a variety of commensal and pathogenic microorganisms has shown that bacteria utilize a variety of mechanisms to modulate the canonical NF-κB pathway^57^. It is possible that butyrate concentrations in the supernatant (which were about 10 times lower than physiological concentrations^58^) helped inhibit the NF-κB pathway^59^. It may also be that other compounds, such as polysaccharides^60^, peptidoglycans^61^, and proteins^62^, were secreted into the supernatant or exposed on the surface of the bacterial membrane^63^. Further research is needed to decipher the underlying mechanisms at work.

We then evaluated how well *C. minuta* could protect epithelial cells from TNF-α-induced permeability using a Caco-2 cell line. We found that the bacterium successfully maintained the integrity of the epithelial cell monolayer following induced inflammation. Individuals with IBDs have very low levels of the adhesion molecules that regulate intestinal permeability^64^; *C. minuta* could help restore proper permeability and limit any damage that has occurred. Recent work has highlighted that *Escherischia coli* Nissle 1917 could attenuate declines in TEER induced by TNF-α and IFNγ, notably by inhibiting the NF-κB-mediated activation of the MLCK-P-MLC signaling pathway^65^. *F. prausnitzii* and *Roseburia intestinalis* have also been found to help reverse impaired epithelial barrier function by modulating the expression of tight junction proteins and decreasing paracellular permeability^66^. It would be worthwhile to decipher the precise mechanism in use by *C. minuta*.

To ascertain whether *C. minuta* displayed the same anti-inflammatory properties in vivo, we performed experiments using two different animal models of colitis: a mouse model of moderate, DNBS-induced colitis and a rat model of severe, TNBS-induced colitis^39^. Based on the macroscopic scores, treatment with *C. minuta* significantly limited colon damage in both models. To characterize the bacterium’s immunomodulatory effects, we assessed neutrophil infiltration in colonic tissues by monitoring MPO activity (in the mice) and LCN-2 levels (in the rats). In both models, the metrics were lower in *C. minuta*-treated animals. Similar studies found comparable effects in a mouse model of TNBS-induced colitis using a treatment based on *Parabacteroides distasonis*^67^ and in a mouse model of DNBS-induced colitis using a treatment based on *F. prausnitizii*^68^ and different *Lactobaccillus* strains^56^. Taken together, these findings demonstrate that using single-strain colitis treatments could be effective^6^. It has been shown that IL-8 secretion induces neutrophil activation in inflammed regions^69,70^. Given that *C. minuta* decreased IL-8 secretion and NF-κB activation in vitro, this signaling pathway could have been involved in the reduction of neutrophil activation. Furthermore, a decrease in IL-1β was seen in the *C. minuta*-treated rats (Fig. 5e). IL-1β signaling is mediated by multiple transcription factors, including NF-κB^71^. It is possible that cytokine release in the TNBS-induced colitis model was partially modulated by *C. minuta*’s secretion of NF-κB inhibitors. A similar mode of action has been seen in *F. prausnitzii* in different colitis models^62^, notably via the release of the microbial anti-inflammatory molecule (MAM). Although the NF-κB signaling pathway serves as a major line of defense against pathogens, it can have deleterious effects when overactivated due to the increased production of proinflammatory cytokines^72^. Consequently, it is important to determine which molecules help control pathway activation so that the development of inflammation can be halted. For example, SCFAs such as butyrate can limit inflammation via their inhibitory action^73^.

In conclusion, our study is the first to show that *C. minuta* displays strong immunomodulatory properties in vitro and in vivo. Our findings open the door to intriguing new research questions. Although additional research is obviously needed to better understand the bacterium’s effects and their underlying mechanisms, our work underscores that *C. minuta* holds promise for treating IBDs and merits further study with a view to developing single-strain biotherapies.

## Methods

### Culturing the bacteria

*Christensenella minuta* (DSM 22607) was cultured in Gifu anaerobic medium (GAM broth, HyServe) in an anaerobic chamber (5%/5%/90% CO_2_, H_2_, N_2_) kept at 37 °C. Granulated agar (15 g/L, Difco) was added when necessary. Bacterial cultures were centrifuged at 2500×*g* and then resuspended in 1X Dulbecco’s phosphate-buffered saline (DPBS, Gibco). We then employed these cultures in the in vitro and in vivo experiments described below. To establish the growth curves, cultures were followed for their entire growth cycle (up to 54 h). We used a spectrophotometer (Ultrospec 10) to measure optical density (OD_600_) and thus estimate bacterial counts. Samples of cultures were collected at different timepoints and centrifuged at 4000×*g* at 4 °C for 15 min. We recovered the supernatants and stored them at − 20 °C until we could measure short-chain fatty acid (SCFA) concentrations.

### Characterizing short-chain fatty acid concentrations

Bacterial supernatants were deproteinized overnight at 4 °C via the addition of phosphotungstic acid (10% [*v/v*]); Sigma). We then centrifuged the resulting samples for 15 min at 12,000×*g*. Concentrations of SCFAs were determined using a gas chromatograph (GC; Agilent 6890 N Network) equipped with a split-splitless injector (GC Agilent 7890B), a flame-ionization detector, and a capillary column (15 m × 0.53 mm × 0.5 µm) packed with SP 1000 (Nukol; Supelco 25,236). The flow rate of hydrogen, the carrier gas, was 10 mL/min; the temperatures of the injector, column, and detector were 200 °C, 100 °C, and 240 °C, respectively. We used 2-ethylbutyrate as the internal standard in our samples and employed a panel of SCFA standards. Two replicates were performed for each sample. We collected the SCFA data and integrated the peaks using the GC’s default software (Agilent). To determine the final concentrations of SCFAs, the supernatants were weighed before and after protein precipitation to obtain the appropriate multiplication factor (i.e., the supernatant to sample mass ratio).

### Assessing oxygen sensitivity

To evaluate *C. minuta*’s sensitivity to oxygen, we used bacteria from the cultures described above^55^. Briefly, we grew *C. minuta* for 48 h in a liquid medium. Then, we took 10-µL samples of different concentrations of the bacteria (range of final concentrations: 10^4^–10^9^ CFU/mL) and deposited them on Petri dishes. The dishes were placed outside of the anaerobic chamber and exposed to oxygen for 2, 4, and 24 h.

### Establishing a metabolic profile

We established a metabolic profile for *C. minuta* using AN MicroPlate™ technology (Biolog) in accordance with the manufacturer’s instructions. Briefly, cultures were streaked twice on Biolog Universal Anaerobe Agar (BUA; Biolog) supplemented with 5% (*w/v*) defibrinated sheep blood (Alliance Bio Expertise). We allowed growth to occur for 4 days at 37 °C under anaerobic conditions. Bacteria were swabbed and transferred into prereduced anaerobic inoculating fluid until 65% transmittance was reached. Then, 100 mL of this bacterial suspension was used to inoculate each well of AN MicroPlates™ under anaerobic conditions. We incubated the plates for 24 h at 37 °C under anaerobic hydrogen-free conditions using a GENbox and anaerobic jar system (bioMérieux). Color shifts in each well were evaluated visually and via optical density measurements made at 590 nm (FLUOstar Omega, BMG Labtech).

### Culturing eukaryotic cells

We obtained the human colon adenocarcinoma cell line HT-29 from the European Collection of Authenticated Cell Cultures (ECACC; Sigma). Cells were grown in McCoy’s 5A medium (Gibco) supplemented with 10% (*v/v*) fetal bovine serum (FBS; Gibco) and 1% (*v/v*) penicillin/streptomycin (P/S; Sigma). The cultures were maintained at 37 °C under conditions of 5% CO_2_ until 80% confluence was reached. We obtained the Caco-2 cell line from the American Type Tissue Collection (ATCC®) and maintained it in Dulbecco’s modified Eagle’s medium (DMEM) supplemented with glutaMAX™ (Gibco), 20% heat-inactivated FBS, and 1% non-essential amino acids (Gibco). Cells were kept at 37 °C under conditions of 10% CO_2_ until 80% confluence was reached.

### Characterizing immunomodulation in HT-29 cells

First, we seeded HT-29 cells into 24-well plates (3 × 10^5^ cells per well). After 24–48 h, confluence was reached, and the complete medium was replaced by a McCoy’s 5A medium supplemented with 2% (*v/v*) FBS. After 24 h, we replaced this medium with McCoy’s 5A medium supplemented with 2% (*v/v*) FBS and TNF-α (5 ng/mL, InvivoGen) to which we added either (1) supernatant obtained from the culture medium during *C. minuta*’s stationary phase (at concentrations of 2%, 10%, or 20%) or (2) bacteria (at MOIs of 10, 50, or 100—ratio of bacteria to eukaryotic cells). PBS glycerol was used as a control. After 6 h of coincubation, supernatants were obtained from the cell cultures and stored at -80 °C until interleukine-8 (IL-8) concentrations could be quantified. The latter process was performed using a Human IL-8 ELISA MAX Standard Set (BioLegend); absorbance was measured at 450 nm using a FLUOstar® Omega microplate reader (BMG Labtech).

### Characterizing immunomodulation in HT-29 cells transfected with a NF-κB luciferase reporter vector

HT-29 cells at a density of 3 × 10^5^ cells per well were reverse-transfected with 200 ng pRelA-luc and 10 ng pRL-TK using X-tremeGENE HP DNA Transfection Reagent (Roche) in 24-well plates. Briefly, we prepared the transfection reagent:DNA complex as follows: we added the appropriate quantity of diluted plasmids to serum and antibiotics-free medium (final volume: 50 µL). The mixture was gently combined, and the transfection reagent was added at a ratio of 3:1. We gently mixed the transfection complex, which was then incubated for 15 min at room temperature. We subsequently seeded the complex with fresh cells (i.e., still in suspension). The plates were incubated for 24 h, and the medium was then remplaced by McCoy’s 5A medium supplemented with 2% (*v/v*) FBS. After 24 h of serum starvation, the medium was removed and replaced by McCoy’s 5A medium supplemented with 2% (*v/v*) FBS and 5 ng/mL of TNF-α (InvivoGen) and either (1) supernatant obtained from the culture medium during *C. minuta*’s stationary phase (concentration: 10%) or (2) bacteria and culture medium (10%) as a control. After 6 h of coincubation, we washed the cells twice with cold 1X PBS and lyzed them via exposure to 50 µL of Passive Lysis Buffer (Promega) for 15 min at room temperature under conditions of gentle shaking. Next, the lysates were transferred to microtubes. The Dual-Luciferase® Reporter Assay System (Promega) was used largely in accordance with the manufacturer’s instructions; a few minor modifications were made. Briefly, we transferred 2 × 20 µL samples of the lysates to white 96-well plates; then, we added 50 µL of LAR II solution to each well. First, we quantified levels of firefly luciferase activity. Then, we added 50 µL of Stop & Glo® Reagent, and we quantified levels of Renilla luciferase activity using a FLUOstar® Omega microplate reader (BMG Labtech). NF-κB activity was quantified via the ratio of firefly activity to Renilla activity.

### Assessing effects on intestinal permeability by measuring transepithelial electrical resistance

We used the Caco-2 cell line to determine whether *C. minuta* could affect the epithelial barrier, as previously described^74^. Briefly, Caco-2 cells were grown on Transwell® inserts. When optimal transepithelial electrical resistance (TEER) values were reached (REMS AutoSampler, World Precision Instruments), fresh DMEM was added. Then, the *C. minuta* treatment (bacteria at MOI 40) or the control (PBS 1X) was applied to the apical compartment of the cells. Three hours later, 100 ng/mL of TNF-α (Peprotech) was added to the basal compartment of the cells. TEER was measured just before and 24 h after the treatments. The results were normalized (i.e., relative to basal TEER).

### Assessing effects on DNBS-induced colitis in mice

We assessed the effects of a *C. minuta* treatment on DNBS-induced colitis in mice. We obtained 40 7-week-old male C57BL/6J mice from the Janvier Lab and maintained them under specific pathogen-free (SPF) conditions in the animal facilities of the French National Research Institute for Agriculture, Food, and Environment (IERP Experimental Unit, INRAE). They were housed in cages of five. Our experiments were performed in accordance with European Union legislation on animal welfare and were approved by COMETHEA, our local committee on animal experimentation (n°16744-201807061805486) and in compliance with the ARRIVE relevant guidelines. After a 7-day acclimation period, the 40 mice were divided into 4 groups (n = 10 mice/group): the vehicle control group (no inflammation; CTRL-Vehicle), the inflamed control group (inflammation induced; DNBS-Vehicle), the treatment group (inflammation induced, treatment with *C. minuta;* DNBS-*C. minuta*), and the anti-inflammatory control group (inflammation induced, treatment with 5-ASA; DNBS-5-ASA). For two weeks, we gave the vehicle and inflamed control mice an oral dose of PBS (150 µL) containing 16% (*v/v*) glycerol and the treatment mice were given an oral dose of *C. minuta* (10^9^ CFU/mL). The anti-inflammatory control mice were given an oral dose of 5-ASA (100 mg/kg; Sigma) from the day of the DNBS injection. Gavages were performed daily until the end of the experiment. Then, we anesthetized the mice using an intraperitoneal injection of 0.1% ketamine and 0.06% xylazine; we subsequently gave them an intrarectal injection of DNBS (175 mg/kg) dissolved in 30% ethanol (*w/v*). The vehicle control group received an intrarectal injection of 30% ethanol. Three days after the injections, the mice were euthanized. During the experiment, we measured body mass daily. Colon microscopic scores (Ameho), macroscopic scores (Wallace), and myeloperoxidase (MPO) activity levels were characterized as previously described^75^.

### Assessing effects on TNBS-induced colitis in rats

We assessed the effects of a *C. minuta* treatment on TNBS-induced colitis in rats. We used Sprague Dawley rats and performed this research at an accredited contract research organization (Intestinal Biotech Development, Lille) in accordance with governmental regulations and in compliance with the ARRIVE relevant guidelines. The rats were divided into 4 different groups. For the first 14 days of the experiment, the vehicle (CTRL-Vehicle) and inflamed control rats (TNBS-vehicle) were gavaged with an oral dose of PBS (150 µL) containing 1% (*v/v*) glycerol. The rats were given an oral dose of *C. minuta* (10^9^ CFU/mL) (TNBS-*C. minuta*); 5-ASA granules, the anti-inflammatory control group, were mixed into the rats’ food. Then, we anesthetized the rats for 2 h and administered an intrarectal injection of TNBS (80 mg/kg) dissolved in 40% ethanol (*w/v*) to induce colitis. The rats were euthanized four days after the injection, and the effects of the treatment and controls were assessed, as was colon mass. During the experiment, we measured body mass daily. Colon microscopic and macroscopic scores (Ameho and Wallace, respectively) were characterized as previously described^40,76^. We quantified inflammation by assessing the levels of the proinflammatory cytokines IL-1β and IL-6 and the anti-inflammatory cytokine IL-10 (eBioscience); the level of lipocalin-2 (LCN-2) (Cliniscience) in the colon was determined using ELISA. Briefly, a 1-cm stretch of the distal colon was recovered and homogenized (50 mg/mL) in Tris–HCl buffer containing protease inhibitors (Sigma) and ceramic beads (diameter: 1.4 and 2.8 mm) using a Precellys tissue homogenizer. Samples were centrifuged for 20 min, and the supernatant was frozen at − 80 °C.

### Statistical analysis

All results were expressed as means ± standard error of the mean (SEM). We performed non-parametric statistical analyses—two-sided Mann Whitney U tests—using GraphPad Prism (v. 8.2.1; GraphPad Software). We employed an alpha level of 0.05.
